# Chiral 1,5,7-triazabicyclo[4.4.0]dec-5-ene (TBD)-Catalyzed Stereoselective Ring-Opening Polymerization of *rac*-Lactide: High Reactivity for Isotactic Enriched Polylactides (PLAs)

**DOI:** 10.3390/polym12102365

**Published:** 2020-10-15

**Authors:** Qaiser Mahmood, Guangqiang Xu, Li Zhou, Xuanhua Guo, Qinggang Wang

**Affiliations:** 1 Key Laboratory of Biobased Materials, Qingdao Institute of Bioenergy and Bioprocess Technology, Chinese Academy of Sciences, Qingdao 266101, China; qaiser@qibebt.ac.cn (Q.M.); zhouli@qibebt.ac.cn (L.Z.); guoxh@qibebt.ac.cn (X.G.); 2 Center of Materials Science and Optoelectronics Engineering, University of Chinese Academy of Sciences, Beijing 100049, China

**Keywords:** chiral TBD, organocatalyst, ring-opening polymerization, high reactivity, stereoselectivity

## Abstract

Chiral 4,8-diphenyl-1,5,7-triazabicyclo[4.4.0]dec-5-ene (DiPh-TBD) was synthesized and applied to a ring-opening polymerization of *rac*-lactide (*rac*-LA). The chiral DiPh-TBD promoted the synthesis of isotactic enriched polylactides (PLAs) with controlled molecular weight and narrow molecular weight distributions under mild, metal-free conditions. When the [*rac*-LA]/[Cat.] ratio was 100/1, full monomer conversion was achieved within only 1 min and a moderate probability of 0.67 *meso* dyads (*P*_m_) was obtained at room temperature. A chain-end control mechanism (CEC) was found to be responsible for the isoselectivity based on the homodecoupled ^1^H NMR spectrum, the chiral HPLC measurement, and kinetic studies.

## 1. Introduction

Compared with many commercial synthetic polymers, polylactides (PLAs) have gained significant recognition due to their divergent properties of biodegradability and biocompatibility, and have therefore found a wide range of applications in textile, pharmaceutical, packing, electronic, and biomedical fields [1,2]. The direct synthesis of isotactic PLAs from low-cost *rac*-lactide has received considerable interest in the last two decades from both academia and industry because of isotactic PLAs’ superior chemical and physical properties compared to their atactic counterpart [3,4,5]. In this field, metal-based catalysts played a dominant role in producing isotactic PLAs with high catalytic activity [6,7,8]. However, the contamination of polymers due to remnants of metal even in ppm limits their application, particularly in biomedical and electronic fields. To overcome these limitations, the emerging organocatalyzed polymerization provides an alternative strategy to produce metal-free PLAs [9,10,11].

**Figure 1:**
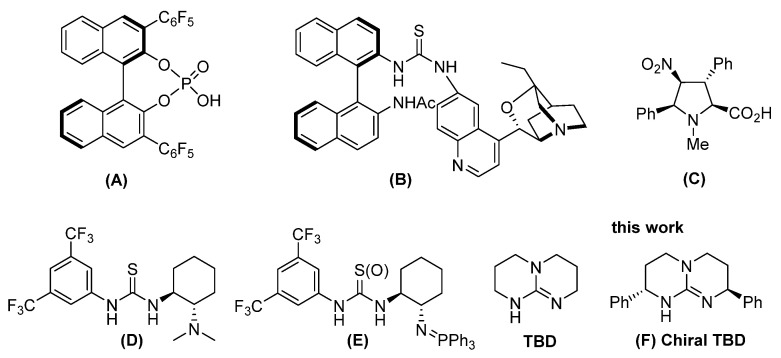
Chiral organocatalysts (**A**–**F**) used for the stereocontrolled ROP of *rac*-LA.

To date, few studies have reported the synthesis of isotactic PLAs directly from *rac*-LA using either achiral or chiral organocatalysts [12,13,14,15,16,17,18,19,20,21,22,23]. Two methods are involved in controlling the stereoselectivity of the ring-opening polymerization (ROP) of *rac*-LA: a chain-end control (CEC) mechanism or an enantiomorphic site control (ESC) mechanism. In the former, the chain end defines the chirality of the subsequent monomer insertion and stereocontrolled PLAs are mainly achieved by enhancing the steric hindrance in achiral catalysts [19]. Notable examples include *N*-heterocyclic carbenes [12,13] and phosphazene superbases [14,15], which produce polymers with a *meso* dyad (*P*_m_) probability in the range of 0.83 to 0.95 at a low temperature between –75 and –70 °C. In an ESC mechanism, the chirality of the catalyst determines the insertion of the next monomer in the growing polymer chain. Terada and Satoh reported the stereocontrolled polymerization of *rac*-LA by chiral binaphthol-derived phosphoric acids (Figure 1A) [16]. A high isoselectivity of *P*_m_ = 0.86 was achieved at 75 °C. A year later, Chen et al. successfully employed β-isocupreidine/thiourea/chiral binaphthylamine (Figure 1B) for the ROP of *rac*-LA and achieved isotactic enriched PLAs with a *P*_m_ of 0.88 at 25 °C [17]. In this field, densely substituted chiral amino acids (Figure 1C) appeared as robust catalysts for the stereocontrolled ROP of *rac*-LA, as reported by Cossío et al. in 2017. These chiral amino acids offered a high isotacticity of *P*_m_ = 0.95 at 25 °C [18]. Taton et al. employed Takemoto’s chiral organocatalyst (Figure 1D) for the asymmetric ROP of *rac*-LA. A high isoselectivity of *P*_m_ = 0.81 (based on CEC) and 0.88 (based on ESC) was achieved [19]. 

We recently investigated bifunctional iminophosphorane-thiourea/urea organocatalysts (Figure 1E) for the ROP of *rac*-lactide [20]. These catalysts showed high stereoselectivity (*P*_m_ = 0.80 according to the CEC mechanism) with controlled molecular weights and distributions. In addition, these catalysts exhibited asymmetric kinetic resolution polymerization of *rac*-LA at –40 °C (*s* factor = 1.6). Most of the aforementioned catalysts require harsh reaction conditions to provide high isotactic PLAs with low reactivity. Therefore, new efficient stereoselective organocatalysts must be designed that can exhibit high stereoselectivity without compromising high reactivity under mild reaction conditions.

In 2006, Waymouth and Hedrick successfully employed a strong organic base, 1,5,7-triazabicyclo[4.4.0]dec-5-ene (TBD), for the ring-opening polymerization of lactide and lactones [24]. However, to obtain stereocontrolled PLAs with TBD, cryogenic conditions were required (*P*_m_ = 0.58 at 23 °C 0.80 at –75 °C) to control the insertion of the monomer and reduce the side reaction (transesterification and epimerization reaction, etc.) [25]. Sterically crowded catalysts with a chiral center offer highly stereoselective polymers in the aforementioned organocatalysts for the stereoselective ROP of *rac*-LA. To further explore these factors, we anticipated that the introduction of a chiral center and bulky group near the active center of TBD would provide a way to control the stereoselectivity of the resultant PLAs with high reactivity (Figure 1F). A detailed study was therefore performed on chiral TBD to ascertain the effects on the polymer structure and the reactivity of the catalyst.

## 2. Materials and Methods 

### 2.1. Materials

All manipulations of air and moisture-sensitive reactions were performed in a glove box and/or using standard Schlenk techniques [26]. *rac*-lactide (99.9%, TCI) was recrystallized three times using toluene, dried under vacuum overnight, and stored in a glove box. Benzyl alcohol (BnOH, 99.9%) was purchased from Alfa Aesar and distilled by cryo-distillation over calcium hydride (CaH_2_). We prepared 1 M of BnOH solution in toluene and used as the initiator. Acetic acid (99.9%, Sigma-Aldrich) solution in dichloromethane was used as a quenching reagent. All of the solvents were dried over sodium metal (toluene and tetrahydrofuran) or CaH_2_ (hexane and dichloromethane) overnight and distilled under inert atmosphere prior to use. ^1^H NMR, ^13^C NMR, and homonuclear decoupled ^1^H NMR spectra were obtained at room temperature using a Bruker Advance spectrometer at 400 MHz and trimethylsilane (TMS) was used as an internal reference. For homonuclear decoupled ^1^H NMR spectroscopic analysis, the relaxation time was measured and fixed at 2.04 s. The molecular weights (*M*_n_s) and molecular weight distributions (MWDs) of the polymers were determined by gel permeation chromatography (GPC, Viscotek VE2001 GPC, Viscotek Corporation, USA) using tetrahydrofuran (THF) as the eluent (flow rate: 1 mL min^−1^ at 40 °C). The sample concentration was 5 mg mL^−1^. The matrix-assisted laser desorption/ionization time of the flight mass spectrometry (MALDI-TOF MS) measurements were obtained using a Bruker BIFLEX III equipped with a 337 nm nitrogen laser. 

### 2.2. General Method of rac-Lactide Polymerization 

In the argon-filled glove box, calculated amounts of lactide, solvent, and initiator were added in sequence to the flam-dried 10 mL reaction tube using a magnetic stirrer. The resulting reaction mixture was stirred for a certain amount time and then polymerization commenced by adding a catalyst (DiPh-TBD) at a fixed temperature. After the required reaction time, the polymerization was quenched by adding 1 M of a solution of acetic acid in dichloromethane. The monomer conversion was determined by performing a ^1^H NMR spectroscopy on the reaction mixture. All the volatiles were removed under reduced pressure and the resulting crude polymer was dissolved in dichloromethane. Precipitation was achieved by adding cold methanol. Dissolution of the polymer and precipitation was repeated three to four times to remove all impurities and the catalyst. The obtained polymer was dried under reduced pressure overnight.

## 3. Results and Discussion

**Scheme 1:**
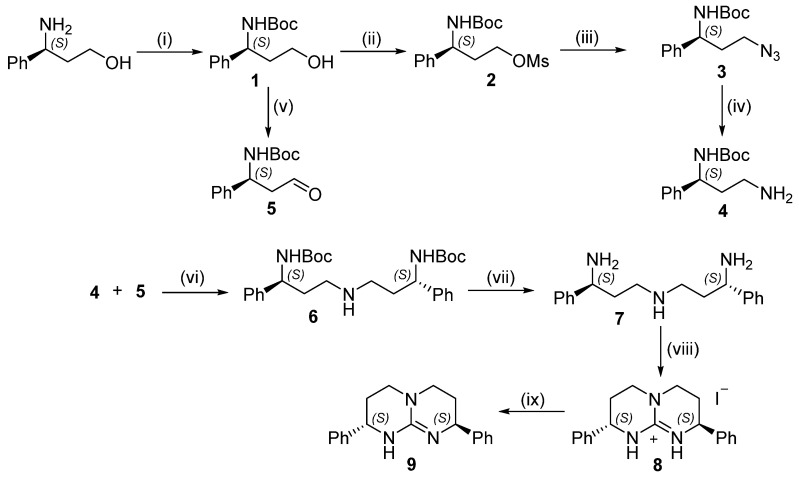
Regents and conditions for synthesis of chiral DiPh-TBD: (i) Et_3_N, di-*tert*-butyl dicarbonate, DCM; (ii) Et_3_N, methanesulfonyl chloride, DCM; (iii) NaN_3_, DMF; (iv) Dry MeOH, Pd/C, H_2_; (v) Et_3_N, dry DCM, dry DMSO, Sulfur trioxide complex, pyridine; (vi) Dry THF, 2 h stirring, then NaBH_4_; (vii) Trifluoracetic acid, stirring; (viii) MeNO_2_, dimethyl trithiocarbonate, stirring, CH_3_COOH, MeI; (ix) NaOH, DCM.

To verify the above hypothesis, chiral 4,8-diphenyl-1,5,7-triazabicyclo[4.4.0]dec-5-ene (DiPh-TBD, Scheme 1, **9**) was designed and synthesized. The detailed synthesis steps are shown in the supporting information. DiPh-TBD was well characterized by ^1^H NMR and ^13^C NMR (Figures S1 and S2, Supplementary Materials). To investigate the catalytic potential of DiPh-TBD for the ring-opening polymerization of lactide, different reaction parameters such as the catalyst-to-initiator ratio, reaction temperature, and solvent effect were systematically studied. The results are presented in Table 1. Preliminary polymerization of *rac*-LA was performed in dry CH_2_Cl_2_ ([LA]_0_ = 1 M) at 25 °C using a [LA]/[Cat.] ratio of 100:1. The monomer conversion was calculated by the relative integration of characteristic methine proton signals in the ^1^H NMR spectrum of the reaction mixture. Generally, BnOH works as an initiator in combination with the catalyst and assists in the initiation of the polymer chain. Therefore, the effect of BnOH was initially studied. With the use of 1 equip of BnOH, monomer conversion reached its highest level of >99% in only 1 min at 25 °C with a molecular weight distribution (PDI) of 1.3 and an average molecular weight of 15,700 g/mol (run 1, Table 1 and Figure S3). More importantly, isotactic-enriched PLAs were obtained with a *P*_m_ of 0.67 (Bernoullian statistics). In comparison with TBD, slightly lower reactivity was shown by DiPh-TBD [24,27], which suggested that increasing steric hindrance near the active site has some influence on the reactivity. When compared with the acyclic guanidine organocatalysts reported by Hecht et al., the reaction time of DiPh-TBD-mediated polymerization for full monomer conversion was much shorter [28]. To study the effect of the catalyst-to-initiator ratio, two more runs of polymerization were performed (runs 2 and 3, Table 1). The use of 2 equiv of BnOH provided quantitative conversion within 1 min again, but a relatively lower molecular weight of PLAs was obtained. It was anticipated that a high concentration of initiator would enhance the chain transfer reaction. Similar findings have been reported elsewhere [20,29]. Without an initiator (BnOH), a longer reaction time of 30 min was required to obtain full conversion (run 3, Table 1). Without using BnOH, polymerization provided PLAs with a comparatively higher molecular weight with an isoselectivity of *P*_m_ = 0.64.

To increase the stereoselectivity, polymerization was performed at a lower temperature. As expected, DiPh-TBD showed lower reactivity; a longer reaction time of 60 min was required to complete full monomer conversion at 0 °C. Isotactic-enriched PLAs with a comparatively higher molecular weight of 19,700 g/mol were obtained. The isoselectivity (*P*_m_) of the obtained polymer improved to 0.69 (run 4, Table 1) [15,20]. In addition to experimenting with polymerization in CH_2_Cl_2_, the effect of other reaction media was also studied under similar conditions. However, all other solvents proved less effective (runs 5 and 6, Table 1). We observed that DiPh-TBD was significantly more active in CH_2_Cl_2_ than in THF and least active in toluene solution (runs 1 vs. 5 vs. 6). Compared to polymerization in CH_2_Cl_2_, 30 min were required for full monomer conversion in THF, whereas 1 h of reaction time was necessary to obtain 100% yield of PLAs in toluene. The PLAs obtained in toluene showed slightly lower isoselectivity of *P*_m_ = 0.62 than in CH_2_Cl_2_. However, those obtained in THF had random microstructure [30]. Among all the solvents, polymerization in CH_2_Cl_2_ provided PLAs with a higher molecular weight with relatively narrow molecular weight distributions.

**Figure 2:**
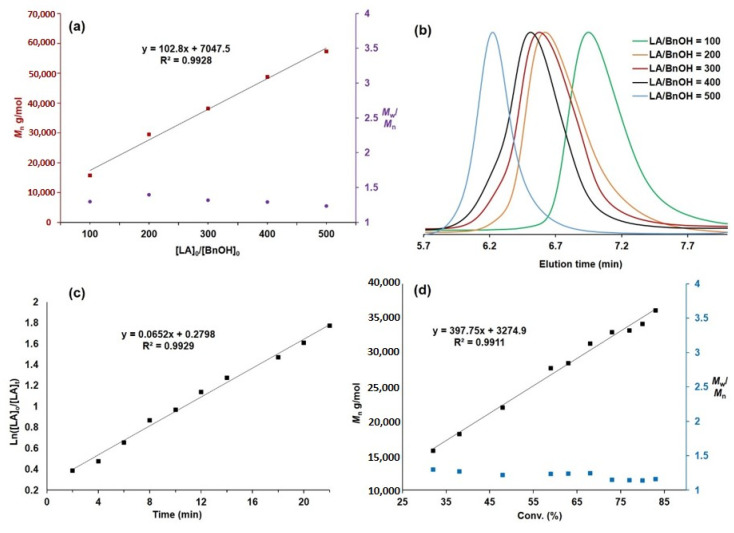
(**a**) Plot of PLAs: *M*_n_(GPC) and dispersity (*M*_w_/*M*_n_) vs. the molar ratio of monomer to initiator ([LA]_0_/[BnOH]_0_) for the polymerization of *rac*-LA (run 1, 7–9, and 13, Table 1). (**b**) GPC traces of the polylactide prepared at different monomer ratios (run 1, 7–9, and 13, Table 1). (**c**) Semilogarithmic plots of ln([LA]_0_/[LA]_t_) vs. time for *rac*-LA polymerization (run 8, Table 1). (**d**) *M*_n_ (GPC) and dispersity (Ð) vs. monomer conversion for PLAs synthesized at room temperature (run 8, Table 1).

Next, DiPh-TBD was exploited for the ROP of *rac*-LA at different feeding ratios of [LA]/[Cat.] (runs 1, 7–9, 13, Table 1). Upon increasing the monomer-to-catalyst ratio, the rate of reaction comparatively decreased. The highest isoselectivity of *P*_m_ = 0.68 was achieved with the [LA]/[Cat.]/[BnOH] ratio of 400/1/1 at room temperature (run 9, Table 1). The average molecular weight consistently increased by increasing the monomer-to-catalyst ratio, while the molecular weight distribution remained unimodal and narrow (PDI = 1.29–1.39), highlighting a characteristic of the living polymerization process (see Figure 2a) [19]. The GPC traces of all PLAs obtained at [LA]/[Cat.] ratios are presented in Figure 2b. In addition, the kinetic study for the [LA]_0_/[Cat.]_0_ ratio of 300 (run 8, Table 1) was also investigated to determine the living polymerization process of the current catalytic system. The first-order kinetic characteristic between ln([LA]_0_/[LA]_t_) and time in the semilogarithmic depiction (Figure 2c, Table S1) and the linear relationship between *M*_n_ and the reaction time (Figure 2d, Table S1) revealed a living chain propagation mechanism, which is further reflected by the low molecular weight distribution of the corresponding polymers [31,32,33]. Furthermore, for the [LA]_0_/[Cat.]_0_ ratio of 500, monomer conversion was determined at different reaction times, such as 15, 30, 45, and 60 min (runs 10–13, Table 1). Maximum conversion was reached after 60 min. Scrutiny of the average molecular weight of the resultant PLAs showed a clear trend of increasing *M*_n_ versus run time, while the *M*_w_/*M*_n_, *P*_m_ properties were found to be similar (runs 10–13, Table 1).

**Figure 3:**
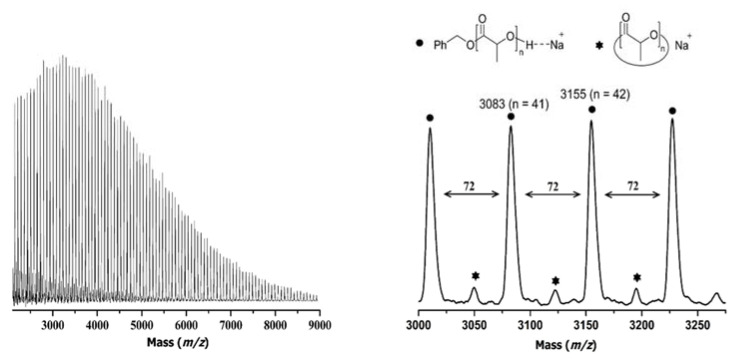
The MALDI-TOF mass spectrum of resulting polylactide using BnOH as an initiator.

The MALDI-TOF study was conducted to determine the end group of the polymer chain and transesterification. As shown in Figure 3, the main series of peaks corresponded to 108 + 23 + 72 × *n*, which showed linear growth of the monomer units with benzyl alcohol as the initiator. In addition, evidence of a small amount of cyclization product was also detected by MALDI-TOF mass analysis. The regular *m/z* = 72 difference between the peaks indicated that significant transesterification took place during the polymerization process, which is consistent with TBD [25]. However, in some cases, transesterification could be used as well. For instance, Hong and coworkers reported a novel transesterification (TEster) strategy to synthesize poly(glycolic acid-co-BL) [34,35]. Another significant application of transesterification is the depolymerization reaction, in which the polyesters are degraded into ester with alcohol to achieve effective recycling of the polymer [36,37].

**Figure 4:**
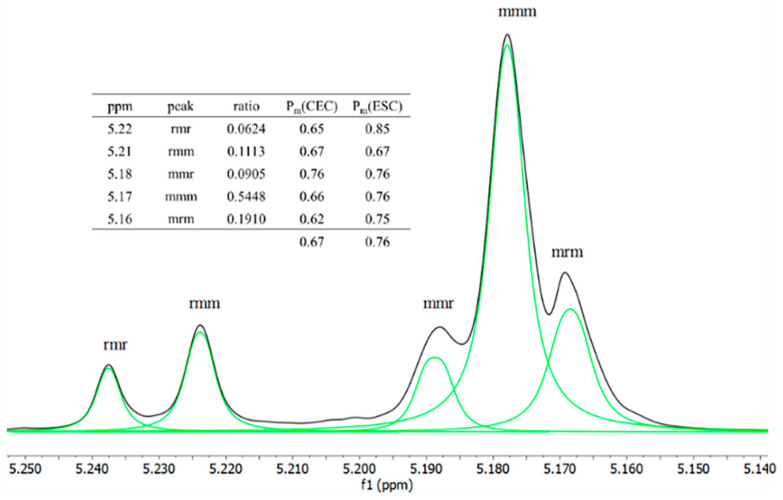
The homodecoupled ^1^H NMR spectrum after deconvolution of the sample (run 7, Table 1).

**Figure 5:**
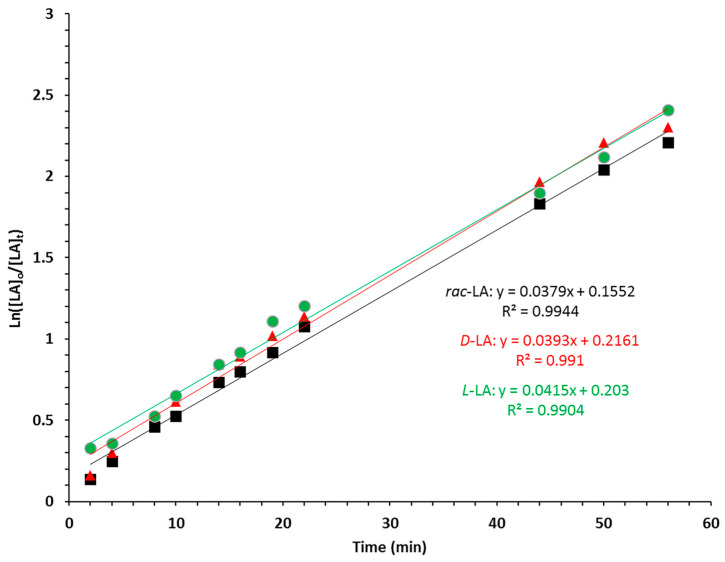
Semilogarithmic plot of ln([LA]_0_/[LA]_t_) *vs.* time for D-LA, L-LA, and *rac*-LA polymerization Table S2).

To verify the control mechanism of this chiral DiPh-TBD, a set of experiments was performed. First, a statistical analysis of the tetrad in the homodecoupled ^1^H NMR spectrum was conducted to identify the possibility of a specific mechanism. According to the homodecoupled ^1^H NMR spectrum from a sample (Figure 4), the *P*_m_ value was calculated as 0.67 and 0.76 with CEC and ESC mechanisms, respectively [38]. If the CEC mechanism is involved exclusively in the current polymerization process, then the ratio of peaks should be [mmr] = [rmm] ≠ [rmr], whereas for the ESC mechanism, statistical distribution of the tetrad should be [rmr] = [rmm] = [mmr] = [mrm]/2. However, the experimental distribution of the tetrad was found to be [rmr] = 0.06, [rmm] = 0.1, [mmr] = 0.09, and [mrm] = 0.19. A chain-end control mechanism was supported by the data. Second, during the polymerization, the unreacted monomers at different conversions were also tested with a chiral HPLC measurement. The 0% ee (enantiomeric excess) indicated that the consumption rates of D-LA and L-LA are the same. Third, kinetic studies of the DiPh-TBD-catalyzed ROP of D-LA, L-LA, and *rac*-LA were performed (Figure 5, Table S2). The enantiopure (D and L) and racemic lactide showed kinetics of first-order dependence. We also observed that the polymerization rates of D-LA and L-LA are very close to each other under identical conditions, and are slightly higher than *rac*-LA. By integrating all these experiments, we confirmed that a chain-end control mechanism (CEC) was responsible for the isoselective polymerizations of *rac*-LA when DiPh-TBD was used as the catalyst.

## 4. Conclusions

The first example of chiral DiPh-TBD-catalyzed stereoselective ring-opening polymerization of *rac*-lactide was achieved. This organocatalyst enables PLAs to be produced with controlled molecular weight, low polydispersity distribution, and a well-defined end group under mild conditions. DiPh-TBD exhibited high reactivity, as it yielded full conversion with a monomer-to-catalyst ratio of 100 within 1 min. The highest isoselectivity (*P*_m_) of 0.68 was achieved for the polymerization of 400 equiv of the monomer at room temperature. A chain-end control mechanism (CEC) was responsible for the isoselective polymerizations of *rac*-LA. Further investigations into more chiral TBD are still in progress in our laboratory.

## Figures and Tables

**Table 1:** The isoselective ring-opening polymerization of *rac*-lactide using DiPh-TBD ^a^.

Run	*rac*-LA/Cat./BnOH	Temp. (°C)	Time (min)	Conv. (%) ^b^	*M*_n_^c^ (g/mol)	*M*_w_/*M*_n_^c^	*P*_m_^d^
1	100/1/1	25	1	>99	15,700	1.30	0.67
2	100/1/2	25	1	>99	10,900	1.47	0.61
3	100/1/0	25	30	>99	17,400	1.75	0.64
4	100/1/1	0	60	>99	19,700	1.62	0.69
5 ^e^	100/1/1	25	30	95	12,100	2.07	0.52
6 ^f^	100/1/1	25	60	>99	15,500	2.14	0.62
7	200/1/1	25	5	>99	29,400	1.39	0.67
8	300/1/1	25	30	>99	38,200	1.32	0.66
9	400/1/1	25	60	>99	48,800	1.29	0.68
10	500/1/1	25	15	29	26,300	1.30	0.64
11	500/1/1	25	30	52	39,500	1.28	0.62
12	500/1/1	25	45	63	47,400	1.28	0.65
13	500/1/1	25	60	95	57,400	1.23	0.63

^a^ Reaction conditions: Polymerizations were conducted in CH_2_Cl_2_ (1 M) at 25 °C unless otherwise stated. ^b^ Measured by ^1^H NMR spectra in CDCl_3_. ^c^ *M*_n_ and *M*_w_/*M*_n_ values were determined by GPC in THF against a polystyrene standard. ^d^ Probability of finding *meso* dyads calculated from the homonuclear decoupled ^1^H NMR spectrum after deconvolution; calculations were based on the CEC mechanism. ^e^ THF (1 M) used as solvent. ^f^ Toluene (1 M) used as solvent.

## Supplementary Materials

The following are available online at https://www.mdpi.com/2073-4360/12/10/2365/s1. Scheme S1: Regents and conditions for synthesis of chiral DiPh-TBD, Table S1: Kinetic studies for the polymerization of *rac*-LA using DiPh-TBD, Table S2: Kinetic studies of the polymerization of *rac*/D/L-LA initiated using DiPh-TBD, Figures S1 and S2: ^1^H and ^13^C NMR spectrum of DiPh-TBD respectively, Figure S3: ^1^H NMR spectrum of polylactide.

## References

[1] Zhang X., Fèvre M., Jones G.O., Waymouth R.M. (2017). Catalysis as an Enabling Science for Sustainable Polymers. Chem. Rev..

[2] Ueda H., Tabata Y. (2003). Polyhydroxyalkanonate derivatives in current clinical applications and trials. Adv. Drug Deliv. Rev..

[3] Natta G., Pino P., Corradini P., Danusso F., Mantica E., Mazzanti G., Moraglio G. (1955). Crystalline high polymers of α-olefins. J. Am. Chem. Soc..

[4] Thomas C.M. (2010). Stereocontrolled ring-opening polymerization of cyclic esters: Synthesis of new polyester microstructures. Chem. Soc. Rev..

[5] Xu G., Mahmood Q., Lv C., Yang R., Zhou L., Wang Q. (2020). Asymmetric kinetic resolution polymerization. Coord. Chem. Rev..

[6] Dijkstra P.J., Du H., Feijen J. (2011). Single site catalysts for stereoselective ring-opening polymerization of lactides. Polym. Chem..

[7] Pilone A., Press K., Goldberg I., Kol M., Mazzeo M., Lamberti M. (2014). Gradient Isotactic Multiblock Polylactides from Aluminum Complexes of Chiral Salalen Ligands. J. Am. Chem. Soc..

[8] Nomura N., Ishii R., Akakura M., Aoi K. (2002). Stereoselective Ring-Opening Polymerization of Racemic Lactide Using Aluminum-Achiral Ligand Complexes: Exploration of a Chain-End Control Mechanism. J. Am. Chem. Soc..

[9] Ottou W.N., Sardon H., Mecerreyes D., Vignolle J., Taton D. (2016). Update and challenges in organo-mediated polymerization reactions. Prog. Polym. Sci..

[10] Dove A.P. (2012). Organic Catalysis for Ring-Opening Polymerization. ACS Macro Lett..

[11] Yuan R., Shou Q., Mahmood Q., Xu G., Sun X., Wan J., Wang Q. (2019). Kinetic Studies on Guanidine-Superbase-Promoted Ring-Opening Polymerization of ε-Caprolactone. Synlett.

[12] Jensen T.R., Breyfogle L.E., Hillmyer M.A., Tolman W.B. (2004). Stereoelective polymerization of d,l-lactide using N-heterocyclic carbene based compounds. Chem. Commun..

[13] Dove A.P., Li H., Pratt R.C., Lohmeijer B.G.G., Culkin D.A., Waymouth R.M., Hedrick J.L. (2006). Stereoselective polymerization of rac- and meso-lactide catalyzed by sterically encumbered N-heterocyclic carbenes. Chem. Commun..

[14] Zhang L., Nederberg F., Messman J.M., Pratt R.C., Hedrick J.L., Wade C.G. (2007). Organocatalytic Stereoselective Ring-Opening Polymerization of Lactide with Dimeric Phosphazene Bases. J. Am. Chem. Soc..

[15] Liu S., Li H., Zhao N., Li Z. (2018). Stereoselective Ring-Opening Polymerization of rac-Lactide Using Organocatalytic Cyclic Trimeric Phosphazene Base. ACS Macro Lett..

[16] Makiguchi K., Yamanaka T., Kakuchi T., Terada M., Satoh T. (2014). Binaphthol-derived phosphoric acids as efficient chiral organocatalysts for the enantiomer-selective polymerization of rac-lactide. Chem. Commun..

[17] Zhu J., Chen E.Y.-X. (2015). From meso-Lactide to Isotactic Polylactide: Epimerization by B/N Lewis Pairs and Kinetic Resolution by Organic Catalysts. J. Am. Chem. Soc..

[18] Sanchez-Sanchez A., Rivilla I., Agirre M., Basterretxea A., Etxeberria A., Veloso A., Sardon H., Mecerreyes D., Cossío F.P. (2017). Enantioselective Ring-Opening Polymerization of rac-Lactide Dictated by Densely Substituted Amino Acids. J. Am. Chem. Soc..

[19] Orhan B., Tschan M.J.-L., Wirotius A.-L., Dove A.P., Coulembier O., Taton D. (2018). Isoselective Ring-Opening Polymerization of rac-Lactide from Chiral Takemoto’s Organocatalysts: Elucidation of Stereocontrol. ACS Macro Lett..

[20] Lv C., Zhou L., Yuan R., Mahmood Q., Xu G., Wang Q. (2020). Isoselective ring-opening polymerization and asymmetric kinetic resolution polymerization of rac-lactide catalyzed by bifunctional iminophosphorane–thiourea/urea catalysts. New J. Chem..

[21] Pratt R.C., Lohmeijer B.G.G., Long D.A., Lundberg P.N.P., Dove A.P., Li H., Wade C.G., Waymouth R.M., Hedrick J.L. (2006). Exploration, optimization, and application of supramolecular thiourea-amine catalysts for the synthesis of lactide (co)polymers. Macromolecules.

[22] Miyake G.M., Chen E.Y.-X. (2011). Cinchona alkaloids as stereoselective organocatalysts for the partial kinetic resolution polymerization of rac-lactide. Macromolecules.

[23] Lim J.Y.C., Yuntawattana N., Beer P.D., Williams C.K. (2019). Isoselective Lactide Ring Opening Polymerisation using [2] Rotaxane Catalysts. Angew. Chem. Int. Ed..

[24] Pratt R.C., Lohmeijer B.G.G., Long D.A., Waymouth R.M., Hedrick J.L. (2006). Triazabicyclodecene: A simple bifunctional organocatalyst for acyl transfer and ring-opening polymerization of cyclic esters. J. Am. Chem. Soc..

[25] Moins S., Hoyas S., Lemaur V., Orhan B., Chiaie K.D., Lazzaroni R., Taton D., Dove A.P., Coulembier O. (2020). Stereoselective ROP of rac- and meso-Lactides Using Achiral TBD as Catalyst. Catalysts.

[26] Tidwell T.T. (2001). Wilhelm Schlenk: The Man behind the Flask. Angew. Chem. Int. Ed..

[27] Lohmeijer B.G.G., Pratt R.C., Leibfarth F., Logan J.W., Long D.A., Dove A.P., Nederberg F., Choi J., Wade C., Waymouth R.M. (2006). Guanidine and Amidine Organocatalysts for Ring-Opening Polymerization of Cyclic Esters. Macromolecules.

[28] Eisenreich F., Viehmann P., Müller F., Hecht S. (2015). Electronic Activity Tuning of Acyclic Guanidines for Lactide Polymerization. Macromolecules.

[29] Kan C., Hu J., Huang Y., Wang H., Ma H. (2017). Highly Isoselective and Active Zinc Catalysts for rac-Lactide Polymerization: Effect of Pendant Groups of Aminophenolate Ligands. Macromolecules.

[30] Klitzke J.S., Roisnel T., Kirillov E., Casagrande O.L., Carpentier J. (2013). Yttrium– and Aluminum–Bis(phenolate)pyridine Complexes: Catalysts and Model Compounds of the Intermediates for the Stereoselective Ring-Opening Polymerization of Racemic Lactide and β-Butyrolactone. Organometallics.

[31] Ikpo N., Hoffmann C., Dawe L.N., Kerton F.M. (2012). Ring-opening polymerization of ε-caprolactone by lithium piperazinyl-aminephenolate complexes: Synthesis, characterization and kinetic studies. Dalton Trans..

[32] Naumann S., Thomas A.W., Dove A.P. (2016). Highly Polarized Alkenes as Organocatalysts for the Polymerization of Lactones and Trimethylene Carbonate. ACS Macro Lett..

[33] Wang X., Liu Y., Li Z., Wang H., Gebru H., Chen S., Zhu H., Wei F., Guo K. (2017). Organocatalyzed Anionic Ring-Opening Polymerizations of N-Sulfonyl Aziridines with Organic Superbases. ACS Macro Lett..

[34] Liu X., Hong M. (2019). Transesterification by air/moisture-tolerant bifunctional organocatalyst to produce ‘nonstrained’ γ-butyrolactone-based aliphatic copolyesters: Turning a bane into a boon. Eur. Polym. J..

[35] Liu X., Hong M., Falivene L., Cavallo L., Chen E.Y.-X. (2019). Closed-Loop Polymer Upcycling by Installing Property-Enhancing Comonomer Sequences and Recyclability. Macromolecules.

[36] Petrus R., Bykowski D., Sobota P. (2016). Solvothermal Alcoholysis Routes for Recycling Polylactide Waste as Lactic Acid Esters. ACS Catal..

[37] Whitelaw E.L., Davidson M.G., Jones M.D. (2011). Group 4 salalen complexes for the production and degradation of polylactide. Chem. Commun..

[38] Mou Z., Liu B., Wang M., Xie H., Li P., Li L., Li S., Cui D. (2014). Isoselective ring-opening polymerization of rac-lactide initiated by achiral heteroscorpionate zwitterionic zinc complexes. Chem. Commun..

