# Supplementary material for: Chiral 1,5,7-triazabicyclo[4.4.0]dec-5-ene (TBD)-Catalyzed Stereoselective Ring-Opening Polymerization of rac-Lactide: High Reactivity for Isotactic Enriched Polylactides (PLAs)
Source: Polymers (Basel). 2020 Oct 15;12(10):2365. doi: 10.3390/polym12102365 (PMC7602607; doi:10.3390/polym12102365)
Supplement: Supplementary file 1 [file polymers-12-02365-s001.pdf]

Supplementary Materials

Chiral 1,5,7-triazabicyclo[4.4.0]dec-5-ene (TBD)-Catalyzed Stereoselective Ring-opening Polymerization of *rac*-Lactide: High Reactivity for Isotactic Enriched Polylactides (PLAs)

Qaiser Mahmood [1], Guangqiang Xu[1, 2,*], Li Zhou[1], Xuanhua Guo[1] and Qinggang Wang[1, 2,*]

[1] Key Laboratory of Biobased Materials, Qingdao Institute of Bioenergy and Bioprocess Technology, Chinese Academy of Sciences, Qingdao, 266101, China

[2] Center of Materials Science and Optoelectronics Engineering, University of Chinese Academy of Sciences, Beijing, 100049, China

Contents

1. Preparation of *S*,*S*-4,8-Diphenyl-1,5,7-triazabicyclo[4.4.0]dec-5-ene catalyst

2. Kinetic studies for ROP of lactide using DiPh-TBD

3. References

1. Preparation of *S*,*S*-4,8-Diphenyl-1,5,7-triazabicyclo[4.4.0]dec-5-ene catalyst

The chiral TBD was prepared according to the similar procedure reported in the literature.[1] The detailed procedure of all the steps are given below.

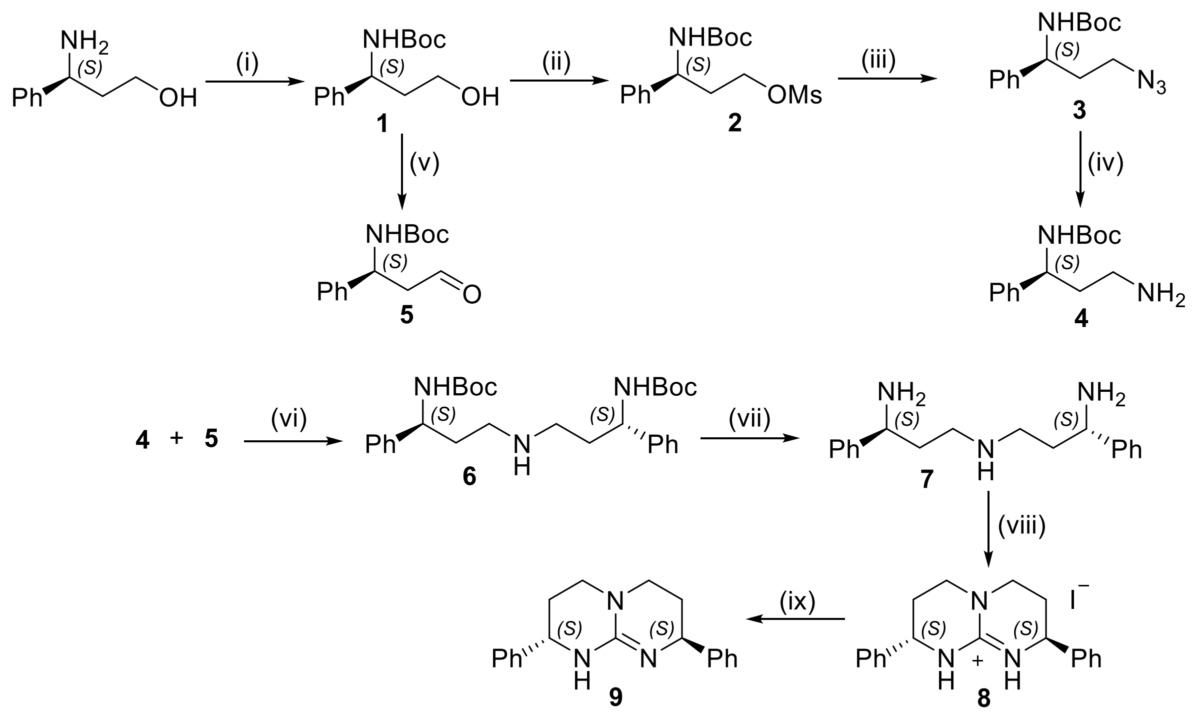

Scheme S1: Regents and conditions for synthesis of chiral DiPh-TBD: (i) Et_3N, di-*tert*-butyl dicarbonate, DCM; (ii) Et_3N, methanesulfonyl chloride, DCM; (iii) NaN_3, DMF; (iv) Dry MeOH, Pd/C, H_2; (v) Et_3N, dry DCM, dry DMSO, Sulfur trioxide complex, pyridine; (vi) Dry THF, 2 h stirring, then $NaBH_4$; (vii) Trifluoracetic acid, stirring; (viii) $MeNO_2$, dimethyl trithiocarbonate, stirring, CH_3COOH, MeI; (ix) NaOH, DCM.

(*S*)-*tert*-Butyl (3-hydroxy-1-phenylpropyl)carbamate (1)

In a round bottom flask, a solution of di-*tert*-butyl dicarbonate (7.22 g, 33.1 mmol) in dichloromethane (20 mL) was added into the solution of (*S*)-3-amino-3-phenylpropan-1-ol (5.0 g, 33.1 mmol) and triethylamine (5.96 mL, 43.0 mmol, 1.30 equiv.) in dichloromethane (50 mL) at 0 °C. The resulting solution was stirred for half hour at 0 °C and continued stirring for two days at ambient temperature. On the completion of reaction, 1 M HCl solution (50 mL) was added and then further stirred for 30 min at room temperature. The organic phase was extracted and washed with water (3 × 20 mL). The organic layers were combined and dried over Na_2SO_4. All the volatiles were removed under reduced pressure affording white solid in quantitative yield and used for nest step without further purification.

(*S*)-3-((*tert*-Butoxycarbonyl)amino)-3-phenylpropyl methanesulfonate (2)

To a stirring solution of **1** (5.0 g, 19.91 mmol) and methanesulfonyl chloride (1.69 mL, 2.50 g, 21.90 mmol, 1.10 equiv.) in dichloromethane (50 mL), triethylamine (3.0 mL, 21.90 mmol, 1.10 equiv.) was added dropwise at 0 °C. The resulting solution was further stirred for overnight at room temperature. All the volatiles were removed and ethyl acetate was then added, washed with 0.5 M H_2SO_4 (20 mL), two times with water (40 mL), brine (40 mL), then dried over sodium sulphate. The resulting solution was concentrated under reduced pressure to afford colorless solid (4.91 g, 75%).

(*S*)-*tert*-Butyl (3-azido-1-phenylpropyl)carbamate (3)

To a solution of **2** (4.0 g 12.15 mmol) in DMF (80 mL), NaN_3 (1.97 g, 30.38 mmol, 2.5 equiv.) was added carefully. The resulting reaction mixture was then stirred for five days at ambient temperature. After removal of all volatiles, dichloromethane was added, washed with brine (3 × 40 mL) and dried over sodium sulphate. The removal of solvent under reduced pressure afforded the colorless solid (3.19 g, 95%).

(*S*)-*tert*-Butyl (3-amino-1-phenylpropyl)carbamate (4)

A solution of **3** (3.0 g, 10.86 mmol) and palladium charcoal (0.5 g of 10% on charcoal) in dry methanol was stirred under 1 atm of H_2 atmosphere at room temperature. After overnight stirring, insoluble particles were filtered through Celite. Evaporation of all the volatiles under reduced pressure and then purification by silica gel column chromatography with an eluent of $MeOH/CH_2Cl_2$ (2:1) afforded a light-yellow solid in good yield (2.17 g, 80%).

(*S*)-*tert*-Butyl (3-oxo-1-phenylpropyl)carbamate (5)

Under inert atmosphere of argon, a suspension of sulfur trioxide pyridine complex (2.54 g, 15.92 mmol, 2 equiv.) and pyridine (1.5 mL) in dry DMSO (3 mL) was added into the solution of **1** (2.0 g, 7.96 mmol, 1 equiv.) and triethylamine (4.4 mL, 31.84 mmol, 4 equiv.) in the mixture of dry DMSO (3 mL) and dry dichloromethane (15 mL) and, stirred for 30 mint at 0 ºC. After two hours stirring at room temperature, 50 mL of water was added at 0 ºC and the aqueous phase was extracted with dichloromethane, washed with brine and the combined layers dried over sodium sulphate. The crude product was then purified by silica gel column chromatography using an eluent of hexane/EtOAc (7:3) which afforded a colorless solid (1.59 g, 80%).

Di-*tert*-butyl ((*1S,1'S*)-azanediylbis(1-phenylpropane-3,1-diyl))dicarbamate (6)

Under argon atmosphere, **4** (1.5 g, 6.0 mmol, 1 equiv.) and **5** (1.5 g, 6.0 mmol, 1 equiv.) were dissolved in dry THF (40 mL) and stirred for one day at ambient temperature. All the volatiles were removed under reduced pressure and then absolute ethanol (40 mL) was added. After stirring for few minutes, $NaBH_4$ (0.7 g, 18 mmol, 3 equiv.) was added to this ethanol solution and stirred for 3 days at room temperature. The solvents were removed and then an aqueous solution of KOH (20%, 40 mL) was added, stirred for half hour, and then extracted with dichloromethane (3 × 50). The combined organic layers were dried over sodium sulphate, filtered and evaporated all the solvents. The purification of resulting residue by silica gel column chromatography (hexane/ethyl acetate: 2/5) afforded white amorphous solid (1.8 g, 62%).

(*S*)-N^1-((*S*)-3-Amino-3-phenylpropyl)-3-phenylpropane-1,3-diamine (7)

To a solution of **6** (1.5 g, 3.1 mmol) in dichloromethane (50 mL), 10 equiv. of trifluoracetic acid (2.37 mL, 31 mmol) was added and stirred for 2 day at room temperature and then heated to reflux for 20 hours. After cooling to room temperature, reaction mixture was washed with 8 M aqueous solution of KOH (50 mL), and extracted with dichloromethane (3 × 50 mL) and dried over sodium sulphate. The evaporation of all the volatile afforded the light-yellow oil in quantitative yield (0.88 g).

Hydroiodide salt of (2*S*,8*S*)-2,8-Diphenyl-2,3,4,6,7,8-hexahydro-1H-pyrimido[1,2-a]pyrimidine （8）

A solution of dimethyl trithiocarbonate (0.49 g, 3.56 mmol, 1.25 equiv.) in nitromethane (4 mL) was added dropwise in a period of 1 hour to the solution of **7** (0.8 g, 2.82 mmol, 1 equiv.) in nitromethane 30 mL) under argon atmosphere at room temperature. The resulting solution was then stirred and heated to reflux for 2 hours. After cooling to the room temperature, acetic acid (0.65 mL, 11.28 mmol, 4 equiv.) and methyl iodide (0.35 mL, 5.64 mmol, 2 equiv.) were added and again heated to reflux for three hours, and then stirred for overnight at room temperature. Following the removal of all the volatiles, water (50 mL) was added and extracted with dichloromethane (3 × 50 mL). The resulting solution was concentrated and eluted on silica gel column with an eluting solvent system of hexane/ethylacetate (1:1) to remove excess reagents. Further elution with methanol/dichloromethane (1:2) provided light red solid (0.85 g, 72%).

(2*S*,8*S*)-2,8-Diphenyl-1,3,4,6,7,8-hexahydro-2H-pyrimido[1,2-a]pyrimidine (9)

To a solution of **9** (0.85 g, 2.03 mmol) in dichloromethane (50 mL), an aqueous solution of NaOH (20 M, 50 mL) was added and then stirred for one hour at room temperature. The organic phase was extracted and washed multiple times with water. The solvents were evaporated and dried over sodium sulphate to obtain the white solid (0.57 g, 96%).

^{1}H NMR (400 MHz, DMSO-d_6, TMS): δ 7.35-7.24 (m, 10H), 4.51 (br, 2H), 3.28-3.23 (m, 2H), 3.08-3.02 (m, 2H), 2.16-2.13 (m, 2 H), 1.86-1.83 (m, 2H). ^{13}C NMR (100 MHz, DMSO-d_6, TMS): δ 150.02, 143.50, 127.60, 126.22, 125.61, 52.05, 44.18, 29.23.

NMR spectra of chiral TBD

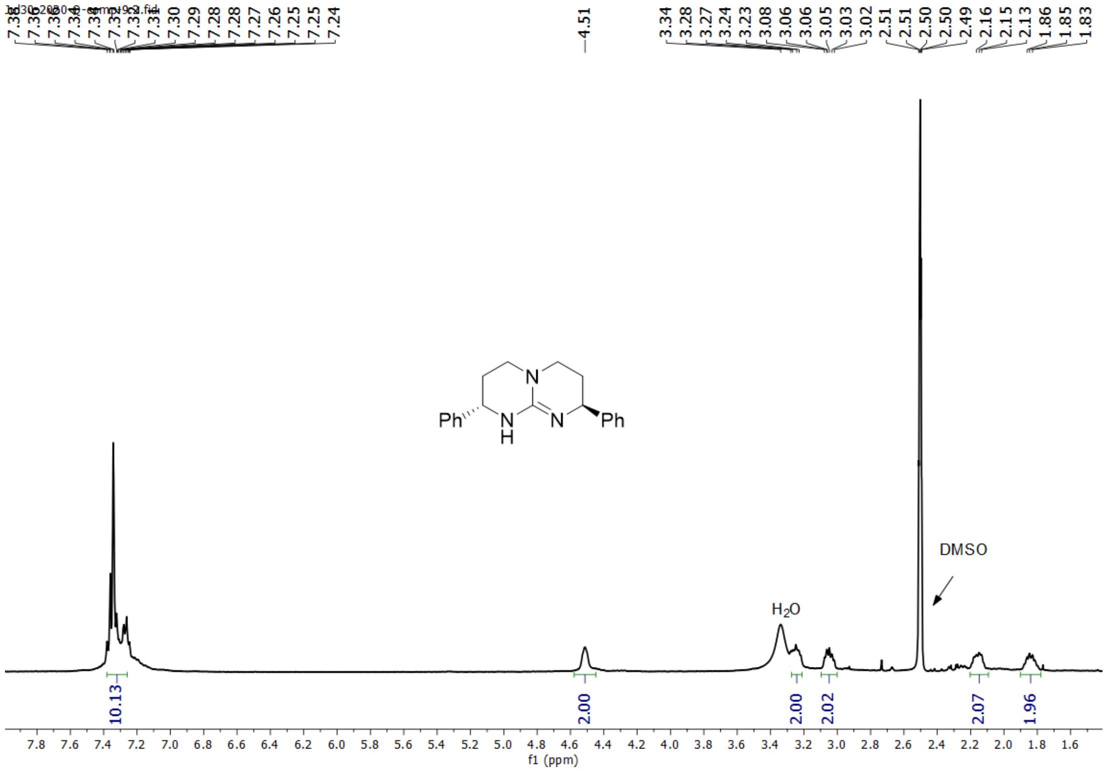

Figure S1 ^{1}H NMR (400 MHz, DMSO-d_6)

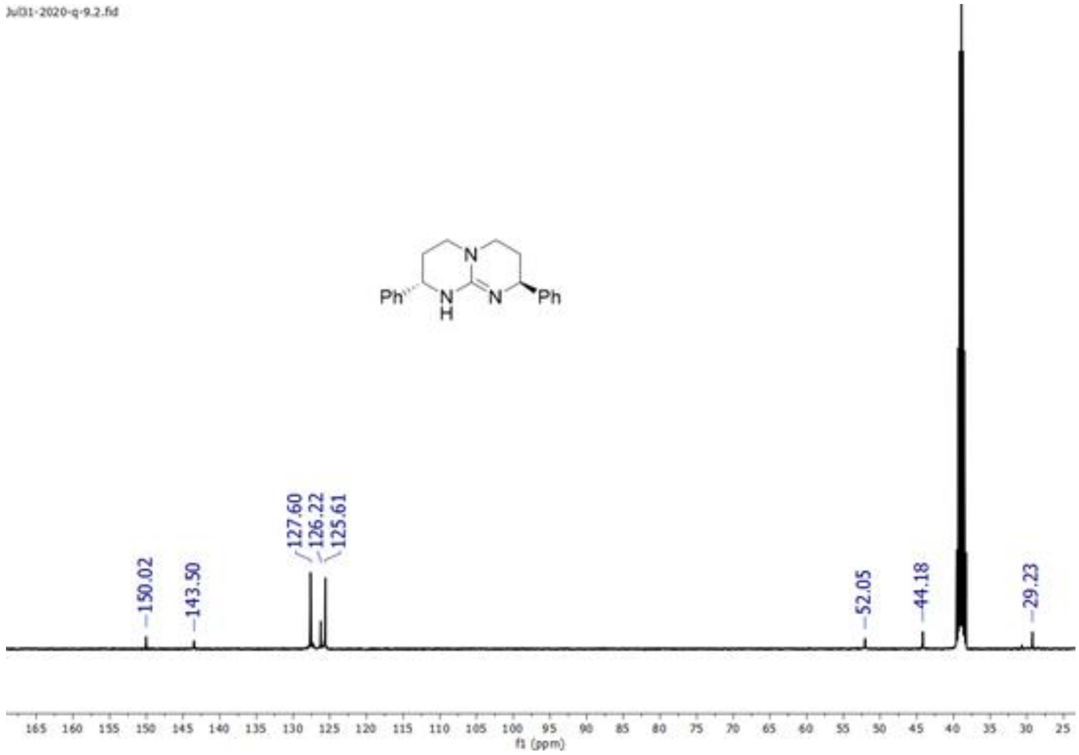

Figure S2 ^{13}C NMR (100 MHz, DMSO-d_6)

2. Kinetic studies for ROP of lactide using DiPh-TBD

Table S1. Kinetic studies for Polymerization of *rac*-LA using DiPh-TBD[a]

Run	Time (min)	Conv. (%)[b]	M_n (g/mol)[c]	M_w/M_n[c]
1	2	32	15,700	1.298
2	4	38	18,100	1.27
3	6	48	22,000	1.22
4	8	58	27,700	1.236
5	10	62	28,400	1.239
6	12	68	31,300	1.248
7	14	72	32,900	1.153
8	18	77	33,200	1.147
9	20	80	34,100	1.14
10	22	83	36,100	1.16

Reaction conditions. [a] Polymerizations were conducted in CH_2Cl_2 (1 M) at 25 ^oC, *rac*-LA/Cat./BnOH: 300/1/1[b] Measured by ^1H NMR spectra in $CDCl_3$. [c] M_n and M_w/M_n values were determined by GPC in THF against polystyrene standard.

Table S2. Kinetic studies of Polymerization of *rac*/D/L-LA initiated using DiPh-TBD[a]

Run	Time (min)	Conv. (%) of *rac*-LA[b]	Conv. (%) of D-LA[b]	Conv. (%) of L-LA[b]
1	2	13	15	28
2	4	22	26	30
3	8	37	41	41
4	10	41	46	48
5	14	52	57	57
6	16	55	59	60
7	19	60	64	67
8	22	66	68	70
9	44	84	86	85
10	50	87	89	88
11	56	89	90	91

Reaction conditions. [a] Polymerizations were conducted in CH_2Cl_2 (1 M) at 25 ^oC, LA/Cat./BnOH: 400/1/1.[b] Measured by ^1H NMR spectra in $CDCl_3$.

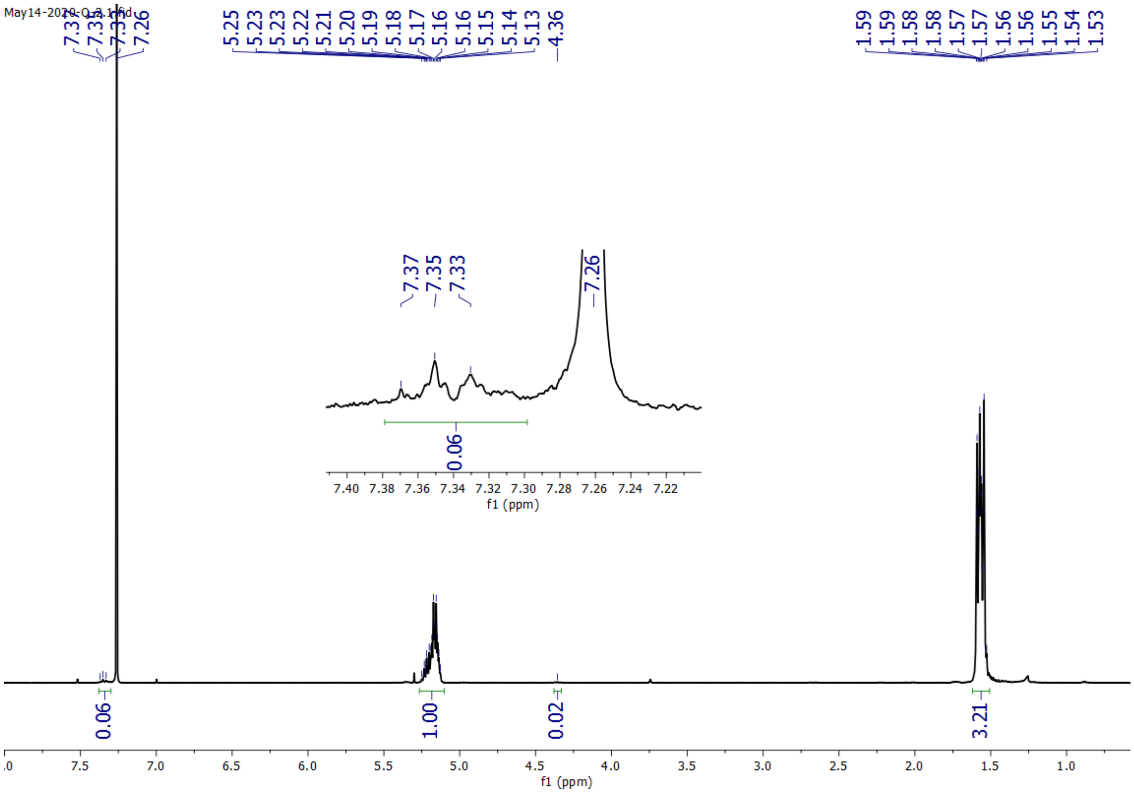

Figure S3. ^{1}H NMR spectrum of polylactide (400 MHz, $CDCl_3$, 298 K) (Table 1, entry 1).

3. References

1) Goldberg, M., Sartakov, D., Bats, J. W.; Bolte M.; Göbel, M. W. *Beilstein J. Org. Chem.* **2016**, 12, 1870.
